# Influence of irrigation during the growth stage on yield and quality in mango (*Mangifera indica L*)

**DOI:** 10.1371/journal.pone.0174498

**Published:** 2017-04-06

**Authors:** Junya Wei, Guoyin Liu, Debing Liu, Yeyuan Chen

**Affiliations:** 1 Applied Science & Technology College, Hainan University, Hainan, China; 2 Tropical Crops Genetic Resources Institute, Chinese Academy of Tropical Agricultural Sciences/ National Tropical Fruit Improvement Center/Hainan Tropical Fruit Cultivation Engineering Technology Research Center, Danzhou, Hainan, China; University of Vigo, SPAIN

## Abstract

Although being one of the few drought-tolerant plants, mango trees are irrigated to ensure optimum and consistent productivity in China. In order to better understand the effects of soil water content on mango yield and fruit quality at fruit growth stage, irrigation experiments were investigated and the object was to determine the soil water content criteria at which growth and quality of mango would be optimal based on soil water measured by RHD-JS water-saving irrigation system through micro-sprinkling irrigation. Five soil water content treatments (relative to the percentage of field water capacity) for irrigation (T1:79%-82%, T2:75%-78%, T3:71%-74%, T4: 65%-70%, T5:63%-66%) were compared in 2013. Amount of applied irrigation water for different treatments varied from 2.93m^3^ to 1.08 m^3^. The results showed that mango fruit production and quality at fruit growth stage were significantly affected under different irrigation water amounts. Variation in soil water content not only had effects on fruit size, but also on fruit yield. The highest fruit yield and irrigation water use efficiency were obtained from the T4 treatment. Irrigation water amount also affected fruit quality parameters like fruit total soluble solids, soluble sugar, starch, titratable acid and vitamin C content. Comprehensive evaluation of the effect of indexs of correlation on irrigation treatment by subordinate function showed that when the soil moisture content were controlled at about 65–70% of the field water moisture capacity, water demand in the growth and development of mango could be ensured, and maximum production efficiency of irrigation and the best quality of fruit could be achieved. In conclusion, treatment T4 was the optimum irrigation schedule for growing mango, thus achieving efficient production of mango in consideration of the compromise among mango yield, fruit quality and water use efficiency.

## Introduction

Worldwide population growth and land use expansion in crops have increased pressure on the availability of water dramatically in the future. Water deficit is the most pervasive impact environmental stress on crop productivity which limits the economical development, especially in China. The increasing water shortage has caused us to investigate the sustainable use of irrigation water. The specific effective water-saving irrigation techniques without detrimentally affecting crop production need to be developed urgently and will be one of the main goals in agricultural production. Micro-sprinkling irrigation has the advantages of both dripping and sprinkling irrigation, when combined with appropriate irrigation schedule, it can effectively reduce water consumption, increase yields and agronomic water use efficiency under conditions of water scarcity [1–3].

Mango (*Mangifera indica* L.) is one of the most important fruit crops in the world and is widely grown in all tropical and subtropical regions including southeastern Asia and Central and South America with a global production of more than 33 million tons [4]. Although being one of the few drought-tolerant plants, mango trees are irrigated to ensure optimum and consistent productivity in China. Fruiting period is considered to be the most sensitive period of water stress, water supply is the most critical during the first six weeks of the fruit developing process, drought will induce late-stage fruit drop and reduced fruit mass via decreased cell size and number [5]. Fruit development takes place inthe natural mango growing season between January and May in Hainan province. Irrigation is necessary because very little rainfall and even drought in the meantime can lead to the decline of yields and quality of the mangoes. Farmers have to irrigate mango trees to ensure high yields and good quality.

Establishing monitoring networks in different crops is presented as a tool for improving agriculture irrigation efficiency. The use of advanced water-saving irrigation techniques and measures can significantly reduce water consumption, improve agronomic water use efficiency, save labor and costs, reduce the adverse effects of agricultural water resource availability from climate change and relieve the crisis of water resource and so on [6]. RHD-JS water-saving irrigation system is an equipment which can achieve the purpose of water-saving irrigation automation through timely monitoring and management of crops irrigation by using the computer, acquisition controller, sensors and other advanced technologies (Ruihua Electronic Co. Handan city, Hebei province China). The main components of RHD-JS water-saving irrigation system include the main control system (computer and control cabinet), electromagnetic valve, soil moisture sensor, weather observation station and other equipments. So far, the RHD-JS water-saving irrigation system has been widely applied in crop irrigation.

In order to investigate the effect of different soil water content during mango fruit growth and development progress, RHD-JS water-saving irrigation system was adopted for real-time monitoring, threshold setting and automatic irrigation in a mango orchard based on antecedent moisture physiological research data [7–9]. Consequently, before using irrigation strategies to regulate mango yield and fruit quality, it is important to obtain adequate information about the relationships between mango yield, fruit quality and water use efficiency. The aims of this study were to provide rationale for water integrated management of mango cultivation and develop a better production system and improve management practice for water resource.

## Materials and methods

### Experimental site

Field experiments were conducted from late February (from flower fading to fruit setting at the initial stage) to early May (fruit maturation stage) in 2013. The experimental site is situated at a mango orchard in the Suburb of Dongfang city, Hainan Province, China (108°36_E, 18°43_N). The area has tropical monsoon climate, annual average temperature of 24.5℃, annual accumulated sunshine hours of 2777.15 h, average annual precipitation of 1150 mm, average annual evaporation of 2596.8 mm, average annual evaporation is greater than the average annual precipitation. The soil is a coastal sandy soil. The organic matter, total nitrogen, available phosphorus, available potassium and soil bulk density in the top soil (0–20 cm) of the experimental plots were 13.44 g.kg^−1^, 9.1 mg.kg^−1^, 48.24 mg.kg^−1^, and 40.0 mg.kg^−1^, respectively according our determination result.

### Experimental design

Irrigation experiments were performed by using 30 healthy and uniform ten-year-old (*Mangifera indica*. cv. Guifei) mango trees grafted on‘Neelum’rootstocks and spaced 3.5 m×4.5 m in the orchard with fertilization and pest control measures were consistent. The mango orchard was managed according to the normal cultural practices in the region. The whole fruit growing season of a mango tree was divided into flower fading to fruit setting stage (stage I), fruit rapidly expanding stage (stage II) and fruit maturation stage (stage III). Irrigation experiments were carried out from February 23 to May 5 according to the fruit development period of mango trees in orchard.

The trees were organized in a randomized block design comprising five repetitive blocks and six treatments of water requirements for micro-sprinkling hoses irrigation which was relative to the percentage of field moisture capacity as a reference to be studied: (T1) replenishment of 79%-82% of field moisture capacity, (T2) replenishment of 75%-78% of field moisture capacity; (T3) replenishment of 71%-74% of field moisture capacity, (T4) replenishment of 67%-70% of field moisture capacity, (T5) replenishment of 63%-66% of field moisture capacity and no irrigation (NI) as the control. Water requirements for irrigation were calculated as soil water content calculated with field moisture capacity as a reference standard.

Soil water content was measured at 30 cm depth from 40cm trunks by the soil moisture sensor of RHD-JS water-saving irrigation system (Ruihua Electronic Co. Handan city, Hebei province China). Daily meteorological data were collected in the nearby automated agrometeorological station of the RHD-JS saving irrigation automation system. This station stores data on air temperature, air relative humidity, windy speed and direction and precipitation. The RHD-JS saving irrigation automation system will begin automatically to practice micro-sprinkling irrigation when the soil water content is reduced to the lowest irrigation thresholds and stops automatically micro-sprinkling irrigation when the soil water content reaches the highest thresholds. Micro-sprinkling is made of thin-walled plastic and has many orifices. The sprinklers of the micro-sprinkling hoses were fixed on the mango tree trunk at a height of 50 cm from the ground with a flow rate of 0.08m^3^/ h and a wetted diameter of 2 m.

For the different treatments, irrigation based on the RHD-JS saving irrigation automation system began on February 23 and ended on May 5. About 45 days after pollination, the *Mangifera indica*. cv. Guifei mango fruits were harvested on May 10. The twelve fruits were selected in four directions of each tree with a tag labeled on each fruit respectively to measure and calculate the fruit growth from early March. Fruit sample length, diameter and single fruit weight were measured once every seven days with Yeyuan Chen method [10], then the flesh samples were homogenized in a blender immediately subjected to a series of tests for the following quality parameters: total soluble solids, titratable acid content, starch content, soluble sugar content and vitamin C content. The total soluble solids were estimated using a hand held refractometer. Total soluble sugar content was determined with anthrone reagent method [11], Titratable acid content, starch content and vitamin C content were measured with Jiankang Cao method [12]. The fruits of each tree of the different irrigation treatments were individually harvested. All the fruits of each tree were weighed and the number of fruits were counted. The average weight of the fruit was determined from these dates in each tree.

Irrigation water use efficiency (Kg/m^3^) was calculated using the following equation [13]:

WUE=Y/amountofirrigationwater×100

Where *WUE* is irrigation water use efficiency (kg m^−3^), Y is total fruit yield (Kg).

### Statistical analysis

Excel 2003 spreadsheet software was used for data processing and mapping. The SAS mixed procedure 9.1.3 version was used to analyze the experimental data, and Duncan’s multiple range test was used to determine significant differences among means. The probability level for determination of significance is 0.05. In the process of data statistics and analysis, the original data were all transformed into relative values. The average subordinate function value of each index was calculated as the comprehensive evaluation standard of different irrigation amount.

## Results

### Meteorological variables

Table 1 presents the average monthly values of air temperature, precipitation and evaporation recorded in the automated agrometeorological station of the RHD-JS saving irrigation automation system from February to May. During the experimental period, the mean daily temperatures were all above 17.2℃, and changed from 23.1 to 29.7℃, and the average temperature gradually increased from February to May during mango fruit growth. The average evaporation of soil moisture increased with the gradually increasing average temperature, and the amount of soil moisture evaporation was much higher than that of precipitation.

**Table 1:** Temperature (T), Evaporation (E) and Precipitation (P) from February to May.

Month	Average temperature (°C)	Maximum temperature (°C)	Minimum temperature (°C)	Evaporation (mm)	Precipitation (mm)
February	23.1	29.7	17.2	122.6	0.4
March	24.8	31.3	15.2	160.6	64.5
April	26.8	33.6	19.5	170.1	4.9
May	29.5	34.8	22.7	218.1	75.6

### Amount of applied irrigation water

**Fig 1:**
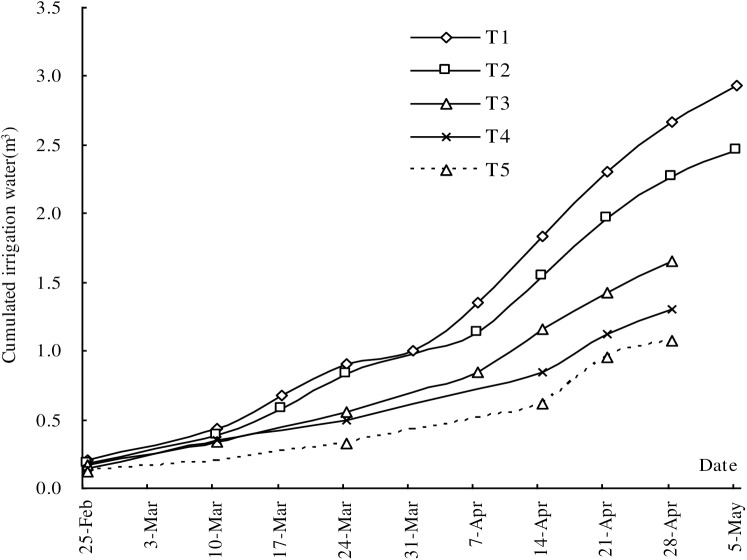
Accumulated irrigation water of different treatments.

During the experimental period from February 23 to May 5, the mango trees received 2.93, 2.46, 1.65, 1.30 and 1.08 m^3^ irrigation water for T1, T2, T3, T4 and T5, respectively (Fig 1,Table 2). The irrigation frequency is not the same for different treatments because of the distinct irrigation thresholds. For T1, T2, T3, T4 and T5, the irrigation times were 10, 9, 7 5 and 5 and the average water use efficiency was 13.54, 16.72, 23.84, 29.41 and 28.45 Kg/m^3^ for these treatments, respectively. For a more detailed analysis of the water applied in the six irrigation treatments, three periods were considered: from flower fading to fruit setting stage (stage I, late February–early April), during fruit rapid expanding stage (stage II, early April–late April) and from the end of the fruit rapid expanding stage until the end of irrigation of season (stage III, late April–late May). Different treatments had the distinct irrigation thresholds (Table 2). In the experimental mango orchard the fruit setting stage was from March 17 to April 7. Fruit rapid expanding stage was from April 7 to April 28. The irrigation water amount for different treatments was different during the fruit development progress. The difference of irrigation water amount between different treatments was very small in the early stage of fruit development. Then the difference gradually increased with the development of fruit and the difference reached the maximum at the fruit maturation stage (Fig 1). This indicated the irrigation water amount was gradually increased with the fruit development in order to meet the water demand for fruit growth. The maximum irrigation amount was during the fruit rapid expanding stage (Table 1).

**Table 2:** Irrigation water applied (mm) in six treatments in the experimental mango orchard from fruit set stage (stage I), fruit rapid expanding stage (stage II), fruit maturation stage (stage III) and seasonal (total).

Treatments	Stage I (m^3^)	Stage II (m^3^)	Stage III (m^3^)	Total (m^3^/tree)
T1	1.00	1.31	0.62	2.93
T2	0.83	1.14	0.49	2.46
T3	0.56	0.87	0.22	1.65
T4	0.50	0.62	0.18	1.30
T5	0.33	0.62	0.13	1.08
NI	-	-	-	-

### Yield and related production functions

Varying soil water contents in different treatments had significant effects on fruit yield showed how the beneficial effect and necessity of irrigation in mango production could be established (Table 3). Total yields ranged from 25.88 to 41.14 kg/tree when the scheduled irrigation thresholds changed between different treatments. As more water was applied, the higher fruit yield was obtained. Total fruit yield was calculated by the average fruit weight (g) and the number of fruit. Irrigation could reduce average single fruit weight, while average yield and number of each mango tree was higher than control, the same with previous studies [14]. The biggest total yield of T2 treatment has the largest number of fruit 193 and the average fruit weight of 213.6g compared with the other treatments. Table 3 indicates that the average fruit weight was weakly correlated with the amount of applied irrigation water. The highest average fruit weight was obtained at NI, 31.8% more than the lowest average fruit weight at T1. The average fruit weight NI was significantly higher than the other treatments and there was no significant difference between the six treatments. Drought in the early stage of the fruit development process caused the increase of fruit dropping which reduced the number of fruit in NI [5]. Of all the treatments, T1 resulted in the lowest average fruit weight. It is consistent with former research [15, 16]. The reasons for higher yields in various treatments can be concluded that the main positive effect of irrigation was on the number of fruit per tree. Irrigation treatments increased fruit yield which was due to a higher crop load rather than larger fruit size. These results are identical with the values of the average weight of the fruit obtained in the harvest by dividing the total weight of the fruits and the number of fruits in each tree (Table 3).

**Table 3:** Some parameters of yield and irrigation under different irrigation treatments.

Parameters	T1	T2	T3	T4	T5	NI
Fruit yield(kg/tree)	39.68±6.45a	41.14±4.64a	39.40±2.59a	38.23±3.36a	30.72±3.17ab	25.88±4.20b
Fruit number(tree^-1^)	188	193	176	157	136	93
Fruit diameter(mm)	53.71±0.99bcd	57.49±3.19abc	54.31±1.53bcd	58.61±4.24a	57.74±2.43ab	50.86±2.77d
Fruit length(mm)	84.23±5.34bc	92.16±8.01b	85.17±2.89bc	102.86±9.18a	88.92±3.15b	79.91±2.83c
Average fruit weight(g)	211.31±23.14b	213.60±23.70b	223.45±14.39b	242.73±18.07b	225.49±21.24b	278.59±14.37a
Irrigation water(tree/m^3^)	2.93	2.46	1.65	1.30	1.08	-
IWUE(kg/m^3^)	13.54b	16.72b	23.84a	29.41a	28.45a	-

Note: In the table lowercase letters difference was extremely significant at 5% level.

**Fig 2:**
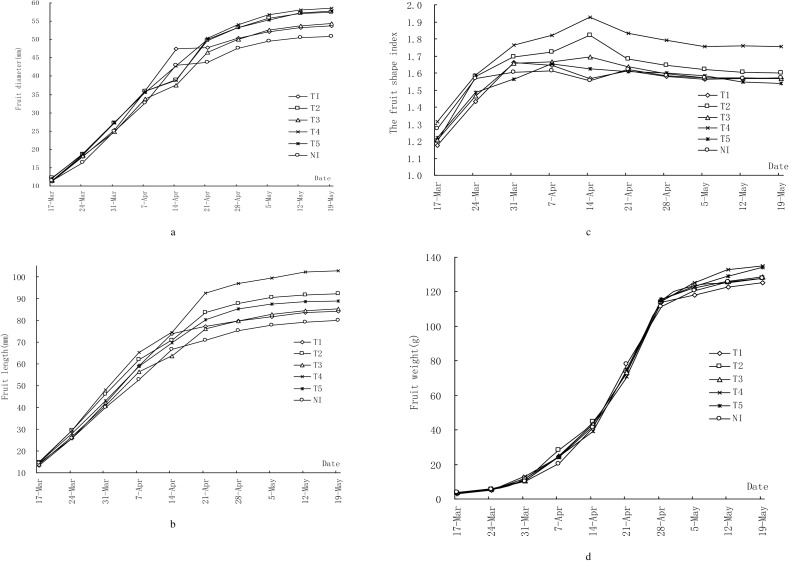
Shows the relationship between fruit diameter (a), fruit length (b), fruit shape index (c) and fruit weight (d) for different treatments.

The difference of fruit yield was mainly related with the number of the fruit and average fruit weight per tree among different treatments. The increase in mango fruit yield in the treatment of T2 compared with the other treatments can be accounted for the average fruit weight and fruit number. According to Table 3, different irrigation thresholds of soil water content not only affected the average fruit weight, but also affected fruit size and performance in fruit diameter and fruit length aspect. Fruit diameter increased rapidly from fruit setting stage to rapid expanding stage (March 17 to April 7) while the change slowed during the fruit maturation stage (after May 5) (Fig 2A). The effect of different soil water content on the increase of mango fruit diameter gradually emerged from April 7. Fruit diameter of non-irrigated treatment (NI) were smaller than the other irrigation treatments during the fruit development period, except April 14. This indicated that lack of irrigation water significantly inhibited fruit diameter increase. The diameter of the fruit in the non-irrigated treatment (NI) was significantly lower than T4 treatment in fruit growth period. Fruit diameter increase rate of T1, T2, T3, T4 and T5 treatment were 5.6%, 13.0%, 6.8%, 15.2%, 13.5% respectively compared with non-irrigated treatment (NI),(Fig 2A). Fruit length followed the same changing pattern as fruit diameter (Fig 2B). Different soil water content treatments had different degrees of impact on fruit diameter and fruit length and T4 treatment had the biggest effects on the increase of fruit diameter and fruit length (Table 3). The change patterns of fruit shape index were gradually increased at early stage, then gradually decreased at latter stage and stabilized after May 12 (Fig 2C), while fruit shape index maximum value appeared at different times for different treatments. The maximum value occurs on April 14 for T2, T3, T4 treatments, April 7 for T5 treatment, March 31 for T1 treatment and non-irrigated treatment (NI). There was no significant difference among different treatments. The reduced weight of the non-irrigated treatment (NI) is brought about by a decrease in water supply which greatly decreases growth. Adequate irrigation can improve mango fruit weight [17].

The change in single fruit weight during fruit growth period followed the typical sigmoid curve and the fruit rapid growth stage was from April 7 to April 28 for all treatments. Flesh weight of single mango fruit increases almost linearly and then tends to slow down (Fig 2D). The fruit weight order of different treatments at harvest was as follows: T4 > T5 >T3 >NI > T2 >T1. The increase in singe fruit weight towards maturity is probably due to an increase in both cell size and intercellular spacing, thus allowing the maximum possible accumulation of assimilates [18].

### Fruit quality

Fruit quality is a major concern for fruit production for its importance to human health and pleasure [19]. Improving mango fruit quality is beneficial to both consumers and growers. The taste and aroma of mango depends on concentration of sugar, total soluble solids, vitamins and amino acids in fruit. Water content, total soluble solids, titratable acid content, starch content, soluble sugar content and vitamin C content of mango fruit in all treatments are listed in Table 4. Variation in soil water content had a significant effect on the nutrition of fruit.

**Table 4:** Effects of different irrigation treatments on fruit quality of mango.

Parameters	T1	T2	T3	T4	T5	NI
Fruit water content (%)	83.40±0.14a	83.17±0.47a	82.70±0.96a	82.32±0.70a	82.67±0.33a	82.73±0.39a
Total soluble solids (%)	8.93±0.13b	10.00±0.27a	10.06±0.54a	10.20±0.34a	10.10±0.33a	10.00±0.21a
Titratable acid (%)	12.20±0.43a	12.00±1.68a	12.14±0.07a	12.94±1.84a	11.47±1.17a	10.95±0.04a
Vitamin C (mg/100g)	33.21±2.09ab	33.55±3.80ab	34.12±0.75a	34.70±0.68a	31.90±0.32b	30.94±0.51b
Soluble sugar (%)	4.08±0.01c	4.29±0.33bc	4.55±0.36bc	4.74±0.70bc	5.35±0.38ab	5.98±0.92a
Starch (%)	10.53±0.14a	10.53±0.16a	10.25±0.31b	9.70±0.33ab	9.87±1.74ab	10.67±0.38a
Comprehensive evaluation	5.999	7.442	7.019	9.264	6.000	4.440

Note: Different letters represent significant differences at P<0.05 according to Duncan’smultiple range tests.

Fruit water content is the maximum proportion and important quality in mango. The highest fruit water content was obtained at T1, but there were no significant differences among these treatments. Total soluble solids are a very important index of quality in mango, either excess or less irrigation water may decrease total soluble solids in the fruit. The highest total soluble solids were obtained at T4, 14.2% more than the lowest total soluble solids at T1, but there were no significant differences among these treatments except T1. It is well known that total tritratable acidity has significant effects on food taste. There is no significant difference for total tritratable acidity content of all treatments. Vitamin C is one of the indispensable components of nutrition in mango fruit. Either excess or less irrigation water may reduce vitamin C in fruit. The highest vitamin C was obtained at T4, 12.2% more than the lowest vitamin C at NI. The results showed that if there was too much or too little water, it would be adverse for the cumulation of vitamin C. The highest soluble sugar was in NI, and it decreased with the decrease in irrigation from T5 to T1. Changes in soluble sugar with soil water content may probably be due to the number of mango water demand and utilization ratio of nutrition under optimum soil water availability. Relative lower soil water content had an advantage on soluble sugar accumulation. Carbohydrate metabolism plays an important role during mango fruit development, particularly changes in starch content. Fruit from the non-irrigated trees had higher starch content than fruit from the irrigated treatments (Table 4). It is consistent with former research [18].

**Fig 3:**
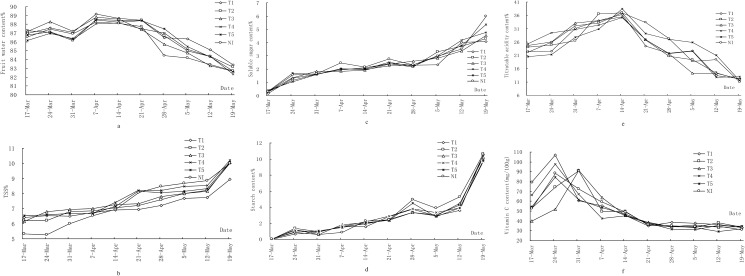
Shows the relationship between fruit water content (a), total soluble solids (TSS) (b), soluble sugar content(c),starch content(d), titratable acid content (e) and vitamin C content (f) for different treatments.

Water seems to have different effects on fruit quality parameters during growth. Fruit water content increased from fruit setting stage to rapid expanding stage and reached the peak at April 7, then decreased slowly during fruit growth and development till fruit maturation (Fig 3A). There is no significant difference for fruit water content of all treatments. Compared with non-irrigated treatment (NI), the increased rate of fruit water content of T1 and T2 treatments were 0.81% and 0.53% respectively, decreased rate of fruit water content of T3, T4 and T5 treatments were 0.03%, 0.50% and 0.07% respectively at harvest. Therefore, adequate irrigation can improve mango water content of fruit. Fruit water content of T3 treatment was the lowest among these treatments when harvested.

Reducing the water content of fruit may be beneficial to the accumulation of dry matter of fruit. According to Fig 3B, fruit soluble solids content gradually increased during fruit growth. Change trends of soluble solid content of fruit were identical between different treatments during the fruit growth process. Fruit soluble solids content of the non-irrigated treatment (NI) was less than other treatments before April 7. But with fruit growth, fruit soluble solids content of non-irrigated treatment (NI) gradually increase and exceed the other treatments. The order of fruit soluble solids content of different treatments when harvest was as follows: T4 > T5 >T3>NI = T2 >T1. There were significant difference between T1 and other treatments. The variation trend of fruit soluble sugar content was also basically identical between different treatments in the early stage of mango fruit growth and there was no significant effect between different treatments (Fig 3C) and the different variation trend gradually become obvious from May. Compared with non-irrigated treatment (NI), decrease rate of fruit water content of T1 to T5 treatment were 31.7%, 28.2%, 23.9%, 20.7%, 10.5% respectively at harvested. Therefore, adequate irrigation can reduce the content of soluble solids and soluble sugar of the fruit.

Change trends of starch content of fruit did not differ across the treatments during the fruit growth process (Fig 3D). The changing amplitude of each treatment was relatively small before April 21. Fruit starch increase amplitude reached its peak at harvest. Therefore, there were no significant differences of fruit starch content between the treatments.

Total tritratable acidity (TTA) increased during the initial stage of fruit growth and decreased at the later stages of fruit growth (Fig 3E). A similar trend has previously been reported in mango fruit [16, 18]. Compared with non-irrigated treatment (NI) fruit, the TTA content increased dramatically in T4. Compared with non-irrigated treatment (NI), increase rate of fruit titratable acid of T4 treatment was bigger than T1, T2, T5 treatment and the effect was amplified by 18.20% at harvest.

Change trends of vitamin C content of fruit were basically identical between different treatments during fruit growth process (Fig 3G) while the peak time was different. Compared with non-irrigated treatment (NI), increase rate of fruit vitamin C content of these treatments were 7.34%, 8.45%, 10.29%, 12.16%, 3.10%. Therefore, adequate irrigation can increase vitamin C content of fruit at harvest.

### Irrigation water use efficiency (WUE)

**Fig 4:**
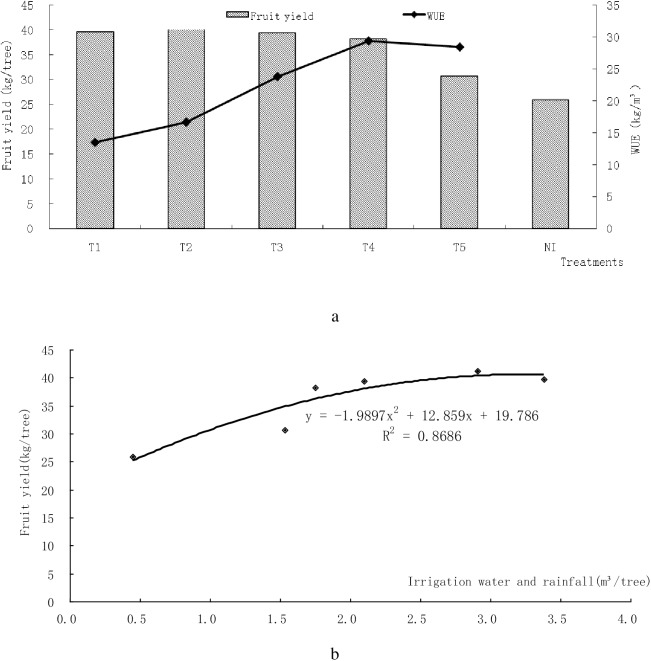
Shows the relationship between fruit yield (a) and irrigation water use efficiency (b) for different treatments.

The irrigation water use efficiency (WUE) is the relation between yield and irrigation water, and was calculated as mango yield divided by amount of irrigation water. Fruit yield and irrigation water use efficiency for different treatments is listed in Table 3 and shown in Fig 4A. The maximum value of WUE, 29.41 kg/m^3^, was determined in T4 whereas minimum value was obtained from T1 with 13.54 kg/ m^3^. As shown in Fig 4A, the average yield of different treatments decreased as the soil water content decreased (from T1 to T5). Irrigation water use efficiency increased across the soil water content treatments (from T1to T5). The maximum yield was obtained in the T2 treatment where the value of WUE was 16.72kg/m^3^. The average yield of T1, T3, T4 and T5 was lower than that of T2 but there were no significant differences among the former four treatments. The average yield of NI was lower than that of each treatment and the difference was significant compared with T1, T2, T3 and T4 treatments, but the difference was not significant between NI and T5 treatments. From Table 3, it can be seen that mango yield was in the order of T2>T1>T3>T4>T5. The maximum value of yield, 41.14 kg/tree, was determined in T2 whereas minimum value was obtained from T5 with 30.72 kg/tree. Using linear regression analysis, the relationship between Fruit Yield and Water Use Efficiency was found to be y = -1.9898x^2^+12.86x+19.785. Fig 4B shows a highly significant determination factor (R^2^ = 0.87) between the relations of mango fruit yield with the total amount of applied irrigation water. The amount of irrigation water has significantly affected fruit production. Production was the highest for the 78–89% soil water content through this linear regression analysis that the mango gained more irrigation water. Consequently, about 2.78 m^3^/tree water is needed for optimal mango growth and production.

**Fig 5:**
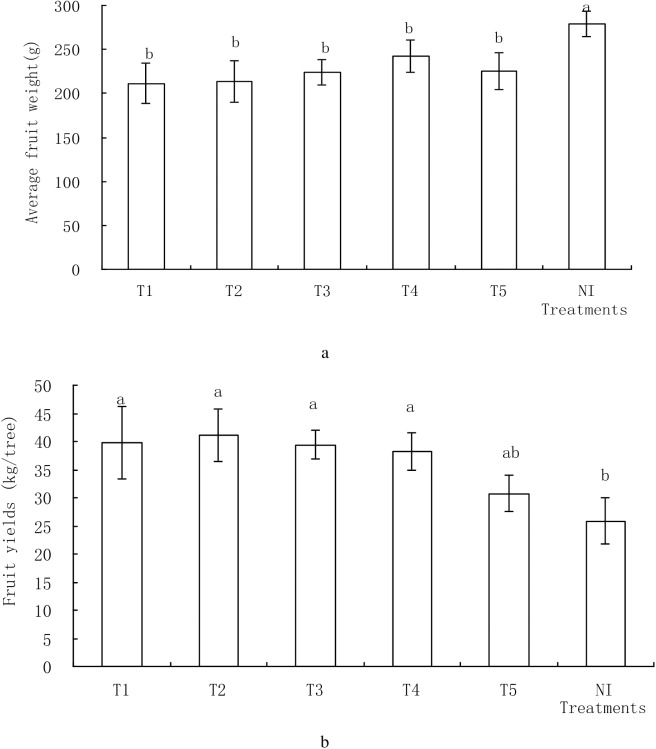
Shows the relationship between average single fruit weight (a) and average yield (b) or different treatments.

According to Fig 5, there were different effect on average single fruit weight (a) and average yield (b) between different treatments. The average yield of T2 treatment was the biggest among these treatments and the number was 41.14 kg/tree. The average yield of non-irrigated treatment (NI) was the smallest among these treatments with 25.88 kg/tree, but offered the biggest average single weights, 278.59 g, similar to the results of previous studies [20, 21]. Compared with non-irrigated treatment (NI), the increase rate of average yield of T1, T2, T3, T4 and T5 treatments were 55.3%, 59.0%, 52.2%, 47.7%, 18.7% respectively, the decrease rate of average single weights of T1, T2, T3, T4 and T5 treatments were 24.15%, 23.33%, 19.79% 12.87% and 19.06% respectively. The total irrigation amount of T4 and T5 treatments was smaller than T1, T2, T3 treatments, but water use efficiency of T4 and T5 treatment were significantly greater than T1, T2, T3 treatments (Table 3). Adequate irrigation could increase average yields but water use efficiency was lower than insufficient irrigation.

There was different effect on each of the irrigation treatments resulting indifferent determination indexs. It was difficult to objectively judge the most suitable soil water content through single determination index in the process of mango fruit growth. Therefore, six treatments of different soil water content were evaluated comprehensively by fruit yield, fruit number, fruit length, fruit diameter, average fruit weight, irrigation water use efficiency and fruit quality using the subordinate function method. Comprehensive evaluation is based on the subordinate function method of fuzzy mathematics theory which transformed the qualitative evaluation into quantitative evaluation, making an overall level of irrigation evaluation of multiple determination index of irrigation. The comprehensive evaluation of the six treatments ranged from T4>T2>T1>T5>T3. So the most suitable irrigation treatment was T4, followed by T2.

## Discussion

Water resource scarcity worldwide makes it necessary to understand the effects of water use reduction in more efficient and competitive crop irrigation. Even though mango is a drought-resistant crop, fruit tree production plays an important role and the efficient use of water resources is mandatory. Cell enlargement and division need adequate water during fruit growth and development period, while lack of water inhibited fruit growth and development [22]. Most of the mango fruit development of natural mango fruits takes place during the dry season and farmers have to irrigate mango trees to ensure high yields and good quality. So soil water management is one of the most important factors for enhancing the yield and quality of mango fruit. In order to achieve more precise management of regulated irrigation in mango production, a better understanding of quantitative relationship between mango yield, fruit quality and water use would be required. The effects of different soil water content on the (*Mangifera indica*. cv. Guifei) mango fruit growth and quality were studied by measuring soil moisture change with soil moisture sensor of RHD-JS in this article. Information about relationship between mango yield, fruit quality and water use efficiency is useful for efficient irrigation management.

Fruit growth is slow at early stage, grows faster at rapidly expansion stage, slowing down at mature stage during the whole fruit growth cycle with one growth peak. Soil water content is essential to the early development of mango fruit. Water supply is the most critical during the first 42 days of fruit development, drought can induce late-stage fruit dropping and reduce fruit mass via decreased cell size and number [5]. There is a negative correlation between the number of fruits on the tree and the average fruit weight. Trends of fruit length, diameter and fruit shape index are identical between different treatments during the fruit development period. Irrigation has no significant effect on the fruit development, similar to the results of previous studies [21, 22].

Fruit soluble solids, soluble sugars and starch content were gradually increasing during fruit growth and development process. There were significant differences of fruit soluble solids content between T3 and T1 treatments. Therefore, adequate irrigation can reduce soluble solids content of fruit through dilution effect of soluble solids [23]. Marcus Nagle et al reported that adequate irrigation can increase mango water content of fruit, dilute soluble solids, increasing water content of fruit can lead to lower soluble solids at harvest [24–26], the same as our result. Titratable acid of fruit of each treatment gradually increases and then gradually decreases during fruit growth process which is similar to those found by Madigu N.O. *et al* [18]. These resultsare similar to previous reports which explain titratable acid increase at initial growth stage and gradual decrease in the late growth stage of mango [27].

Fruit quality is affected by different irrigation treatments. The average fruit weight in the T4 treatment is significantly higher than the other treatments. These results were confirmed with the fruit diameter and length data. For the whole studied period (late February to late May), the average fruit diameter and length of the T4 treatment was significantly higher than that of the other treatments. In the whole period of fruit growth, the titratable acid of the fruit in the T4 treatment was significantly higher than that of the other treatments. The starch and the water content of the fruit juice were not affected by the irrigation treatments. These indicate that different irrigation can cause alteration in the yield and quality whereas adequate water can cause an increase in the average fruit size and an increase in soluble protein and titratable acid.

## Conclusion

In summary, the results for the study period showed that differences in fruit yield and quality production between different irrigation treatments were insignificant. However, the T4 treatment produced significant increases in the average fruit weight, diameter and length and significant increases of the soluble protein and titratable acid in relation to the other treatments. Irrigation of mango plantations is necessary to ensure high fruit yields and a favorable fruit size distribution. Comprehensive evaluation of the correlation on irrigation treatment by subordinate function shows that, when the soil moisture content is controlled at about 65–70% of the field water moisture capacity, water demand in the growth and development of mango can be ensured, and maximum production efficiency of irrigation and the best quality of fruit can be achieved. From these studies, we conclude that T4 treatment, 65%-70% of field water capacity, can promote fruit growth and development and improve fruit internal quality.

## Supporting information

S1 Dataset (PDF)
