# Supplementary material for: Influence of irrigation during the growth stage on yield and quality in mango (Mangifera indica L)
Source: PLoS One. 2017 Apr 6;12(4):e0174498. doi: 10.1371/journal.pone.0174498 (PMC5383426; doi:10.1371/journal.pone.0174498)
Supplement: S1 Dataset — (PDF) [file pone.0174498.s001.pdf]

The Data corresponding to Fig 1

	February 25	March 3	March 10	March 17	March 24	April 7	April 14	April 21	April 28	May 5
T1	0.20	0.43	0.67	0.90	1.00	1.35	1.83	2.31	2.67	2.93
T2	0.18	0.39	0.58	0.83	0.98	1.13	1.55	1.97	2.27	2.46
T3	0.17	0.34	0.45	0.56	0.70	0.84	1.16	1.43	1.65	
T4	0.15	0.35	0.43	0.50	0.61	0.72	0.84	1.12	1.30	
T5	0.12	0.19	0.26	0.33	0.42	0.51	0.61	0.95	1.08	

The Data corresponding to Fig 2（a）

	March 17	March 24	March 31	April 7	April 14	April 21	April 28	May 5	May 12	May 19
T1	11.32	18.06	25.29	36.06	47.39	47.79	50.41	52.15	53.28	53.71
T2	12.22	18.65	27.27	35.95	38.91	49.77	53.19	55.84	57.03	57.49
T3	11.40	18.19	24.74	33.85	37.49	46.47	50.03	52.57	53.74	54.31
T4	11.39	18.57	27.17	35.86	38.70	50.39	54.06	56.77	58.15	58.61
T5	12.09	18.91	27.44	35.57	42.83	49.97	53.32	55.27	57.26	57.74
NI	11.42	16.47	24.98	32.68	42.89	43.82	47.64	49.56	50.56	50.86

The Data corresponding to Fig 2（b）

	March 17	March 24	March 31	April 7	April 14	April 21	April 28	May 5	May 12	May 19
T1	13.29	25.90	42.02	59.24	73.78	77.11	79.69	81.63	83.59	84.23
T2	14.67	29.46	46.24	61.95	70.78	83.69	87.66	90.52	91.67	92.16
T3	13.78	26.57	40.92	56.32	63.57	76.02	79.83	82.71	84.35	85.17
T4	14.96	29.45	47.97	65.39	74.52	92.42	96.90	99.54	102.18	102.86
T5	14.74	28.05	42.96	58.82	69.60	80.25	85.25	87.43	88.58	88.92
NI	14.47	25.74	40.00	52.66	66.58	70.94	75.35	77.70	79.24	79.91

The Data corresponding to Fig 2（c）

	March 17	March 24	March 31	April 7	April 14	April 21	April 28	May 5	May 12	May 19
T1	1.17	1.43	1.66	1.65	1.57	1.61	1.58	1.57	1.57	1.57
T2	1.20	1.58	1.70	1.72	1.82	1.68	1.65	1.62	1.61	1.60
T3	1.21	1.47	1.66	1.67	1.70	1.64	1.60	1.57	1.57	1.57
T4	1.31	1.59	1.76	1.82	1.93	1.83	1.79	1.75	1.76	1.76
T5	1.22	1.48	1.57	1.65	1.63	1.61	1.60	1.58	1.55	1.54
NI	1.27	1.57	1.60	1.61	1.55	1.62	1.58	1.57	1.57	1.57

The Data corresponding to Fig 2（d）

	March 17	March 24	March 31	April 7	April 14	April 21	April 28	May 5	May 12	May 19
T1	3.08	5.08	11.65	24.25	43.42	73.20	113.66	118.16	122.74	125.44
T2	3.54	5.63	11.88	28.21	44.85	73.77	113.71	123.44	125.44	127.71
T3	3.83	5.91	10.36	24.98	43.56	73.54	115.75	122.10	126.17	128.65
T4	3.24	4.94	12.96	24.02	42.09	70.98	114.18	125.16	132.94	135.09
T5	3.58	5.53	10.75	24.55	39.03	75.07	115.45	122.96	129.24	133.96
NI	3.77	5.64	10.27	20.44	41.30	77.94	111.25	120.56	125.50	127.81

The Data corresponding to Fig 3（a）

	March 17	March 24	March 31	April 7	April 14	April 21	April 28	May 5	May 12	May 19
T1	86.96	87.58	87.11	89.18	88.69	88.47	86.49	86.35	85.10	83.40
T2	87.25	87.08	86.18	88.53	88.48	88.47	86.68	84.96	84.35	83.17
T3	87.18	88.28	87.22	88.81	88.59	87.40	85.71	84.74	83.32	81.70
T4	86.77	87.16	86.28	88.07	88.14	87.50	87.02	85.15	84.38	82.33
T5	86.19	86.97	86.37	88.51	88.22	88.40	87.48	85.45	84.33	82.67
NI	86.58	87.43	86.93	88.24	88.10	87.76	84.43	84.17	83.44	82.73

The Data corresponding to Fig 3（b）

	March 17	March 24	March 31	April 7	April 14	April 21	April 28	May 5	May 12	May 19
T1	6.4	6.5	6.5	6.7	6.9	6.9	7.2	7.7	7.7	8.9
T2	6.2	6.2	6.8	6.9	7.1	7.2	7.6	8.0	8.2	10.0
T3	6.1	6.8	6.9	7.0	7.3	7.3	7.8	8.0	8.1	11.1
T4	6.5	6.6	6.7	6.7	7.4	8.2	8.2	8.5	8.5	10.2
T5	6.5	6.6	6.7	6.7	7.1	8.1	8.1	8.1	8.3	10.4
NI	5.3	5.3	6.0	6.5	6.9	8.1	8.5	8.7	8.9	10.0

The Data corresponding to Fig 3（c）

	March 17	March 24	March 31	April 7	April 14	April 21	April 28	May 5	May 12	May 19
T1	0.1737	1.3208	1.6200	2.0147	1.9624	2.2351	2.2993	2.3128	4.0218	5.9814
T2	0.3139	1.1436	1.6146	1.9386	2.1122	2.3939	2.1672	3.3005	3.7153	5.3521
T3	0.3086	1.3356	1.8344	1.8272	1.9220	2.3872	2.5998	2.8090	3.3563	4.5492
T4	0.4200	1.0352	1.5871	2.0341	1.9941	2.5095	2.2099	3.0214	4.1723	4.2966
T5	0.3401	1.7024	1.6123	1.9956	2.0311	2.3999	2.3337	2.9213	3.7783	4.0844
NI	0.3014	1.5584	1.6433	2.4712	2.1698	2.7648	2.3129	2.9706	3.4877	4.7445

The Data corresponding to Fig 3（d）

	March 17	March 24	March 31	April 7	April 14	April 21	April 28	May 5	May 12	May 19
T1	0.02	0.64	0.93	1.63	1.57	2.83	3.74	2.95	3.59	10.53
T2	0.02	1.39	0.97	1.56	1.93	2.47	3.33	3.05	4.24	10.53
T3	0.02	1.20	0.59	1.82	2.09	2.37	3.36	2.94	4.43	10.25
T4	0.07	1.13	0.93	1.60	2.16	2.89	3.80	3.32	3.89	9.70
T5	0.03	0.91	1.07	1.46	2.02	2.36	4.50	2.82	3.91	9.87
NI	0.04	0.88	0.63	0.89	2.26	2.44	4.95	3.92	5.26	10.67

The Data corresponding to Fig 3（e）

	March 17	March 24	March 31	April 7	April 14	April 21	April 28	May 5	May 12	May 19
T1	22.38	22.58	31.12	34.01	36.25	24.45	21.74	22.66	13.40	12.20
T2	24.04	24.80	26.51	36.51	36.87	26.75	20.58	19.40	14.45	12.00
T3	24.73	25.96	33.08	34.01	35.39	26.88	21.53	14.25	14.32	12.14
T4	25.36	29.46	30.73	32.34	35.09	26.86	21.71	22.61	12.69	12.94
T5	20.54	21.29	27.80	30.82	38.47	29.11	27.04	25.79	21.01	11.47
NI	22.19	25.83	32.12	33.13	36.93	33.32	27.24	18.77	19.35	10.95

The Data corresponding to Fig 3（f）

	March 17	March 24	March 31	April 7	April 14	April 21	April 28	May 5	May 12	May 19
T1	79.42	107.04	60.77	55.50	45.47	35.04	38.68	37.43	36.36	33.21
T2	53.06	74.29	90.92	49.69	49.69	35.72	34.27	33.51	38.19	33.55
T3	39.92	51.75	91.37	63.49	45.57	38.33	34.26	36.09	33.78	34.12
T4	66.49	97.77	67.11	42.58	45.25	37.60	31.65	31.21	35.32	34.70
T5	54.26	84.25	61.32	53.36	46.96	37.49	34.75	33.43	29.38	31.90
NI	52.58	88.58	72.63	59.15	48.93	36.22	35.10	34.00	34.35	30.94

The Data corresponding to Fig 4（a）

	Fruit yield	WUE
T1	39.68	13.540
T2	41.14	16.720
T3	39.40	23.840
T4	38.23	29.410
T5	30.73	28.450
NI	25.88	

The Data corresponding to Fig 4（b）

	Irrigation water and rainfall	Fruit yield
T1	3.38	39.68
T2	2.91	41.14
T3	2.10	39.40
T4	1.75	38.23
T5	1.53	30.72
CK	0.45	25.88

The Data corresponding to Fig 5（a）

	T1	T2	T3	T4	T5	NI
Fruit yield	39.68	41.14	39.40	38.23	30.72	25.88

The Data corresponding to Fig 5（b）

	T1	T2	T3	T4	T5	NI
Average single fruit weight	211.31	213.60	223.45	242.73	225.49	278.59
